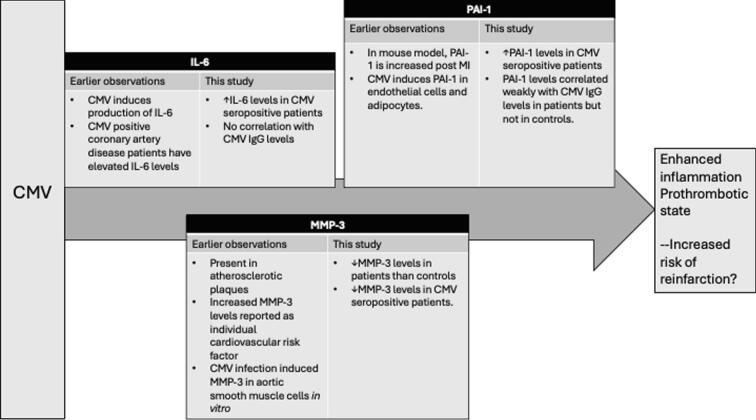# Enhanced levels of IL-6 and PAI-1 and decreased levels of MMP-3 in cytomegalovirus seropositive patients with prior myocardial infarction

**DOI:** 10.1016/j.ijcha.2024.101570

**Published:** 2024-12-02

**Authors:** Xinling Xu, Angela Silveira, Pia Lundman, Afsar Rahbar, Cecilia Söderberg-Nauclér

**Affiliations:** a Department of Medicine Solna, Unit Microbial Pathogenesis, Karolinska Institutet, Stockholm, Sweden; b Department of Infectious Diseases, Karolinska University Hospital, Stockholm, Sweden; c Department of Medicine Solna, Division of Cardiovascular Medicine, Karolinska Institutet, Stockholm, Sweden; d Division of Cardiovascular Medicine, Department of Clinical Sciences, Danderyd Hospital, Karolinska Institute, Stockholm, Sweden; e Department of Clinical Neurology, Karolinska University Hospital, Stockholm, Sweden; f Department of Biosciences, InFLAMES Research Flagship Center, MediCity, University of Turku, Finland

**Keywords:** Cytomegalovirus, Inflammation, Cytokines, Atherosclerosis, Myocardial infarction

## Abstract

**Background:**

Efforts to understand atherosclerosis, a major cause of ischemic heart disease, have linked several lifestyle factors to increased risk for developing cardiovascular disease. Some studies suggest that cytomegalovirus (CMV), a widely prevalent herpesvirus, is reactivated in atherosclerotic plaques and associated with higher cardiovascular mortality risk. We aimed to explore whether CMV seropositivity and CMV-IgG antibody levels correlate with relevant biomarkers in a cohort of patients with myocardial infarction (MI) and matched controls.

**Methods and results:**

We analyzed a dataset from 324 survivors of MI treated in Stockholm between 1996 and 2001. Blood samples collected three months after MI were used to measure protective Apo B100 autoantibodies, metabolic, and inflammatory biomarkers. CMV serology was performed on stored serum samples. Correlation analyses were conducted between biomarkers and CMV serostatus in 324 patients and age- and sex-matched controls. While CMV seroprevalence was equal, the CMV-IgG levels were higher in controls. Among various factors examined, CMV seropositive MI patients had elevated levels of plasminogen activator inhibitor-1 (PAI-1) and interleukin-6, along with lower levels of MMP-3, than CMV seronegative MI patients. CMV-IgG levels correlated positively with PAI-1 levels in patients. Although CMV seropositivity was associated with increased proinsulin levels, there was no correlation with diabetes diagnosis.

**Conclusions:**

Our findings suggest an enhanced inflammatory and prothrombotic state in CMV seropositive patients after MI. Notably, patients had lower levels of CMV IgG than controls.

## Highlights

• Equal CMV seroprevalence in MI patients and controls.
• CMV seropositive patients had higher levels of PAI-1 and IL-6 compared to −negative.
• Weak positive correlation between CMV IgG levels and PAI-1 in MI patients.
• CMV seropositivity was associated with higher proinsulin but not diabetes diagnosis.
• Results suggest an enhanced inflammatory state in CMV seropositive patients after MI.

## 1 Introduction

Ischemic heart disease is caused by formation of atherosclerotic plaques in arteries containing low-density lipoprotein (LDL) particles and inflammatory cells. The pathogenesis of atherosclerosis and myocardial infarction (MI) is multifactorial; known contributors include dyslipidemia, hypercholesterolemia, diabetes, hypertension, inflammatory and immunologic factors, plaque rupture, thrombosis, and smoking. Many of these risk factors also contribute to endothelial dysfunction, including accumulation and oxidation of LDL particles in the vascular wall. This results in activation of the adaptive immune system targeting neo-epitopes formed or exposed as a result of the oxidation process [1], [2], [3], [4], [5]. Apolipoprotein B100 (apoB100) is the protein component of LDL. Previous studies have identified important epitopes of apoB100 that are recognized by autoantibodies, among which both native and malondialdehyde (MDA)-modified apoB100 peptides can be found [6]. Björkbacka et al. [7] reported that lower levels of certain apoB100 autoantibodies are associated with increased risk of coronary events.

Human cytomegalovirus (CMV) is a species-specific and ancient betaherpesvirus that has co-evolved with its human host through the ages; its unique and sophisticated ways of evading our immune system allow for lifelong latency and persistence, without any known harmful effects for the immunocompetent host. The mean seroprevalence of CMV in Sweden is 71–82.3 % [8], [9] among adults. CMV is transmitted with bodily secretions, such as breast milk and saliva. As a result, CMV infection is often acquired in early childhood. While usually asymptomatic in immunocompetent hosts, CMV infection can prove fatal to immunocompromised individuals, such as transplant recipients and acquired immunodeficiency syndrome (AIDS) patients. Latent CMV is reactivated by immune system activation, i.e., any instance of inflammation; trauma [10], autoimmune diseases, and cancer [11], [12], [13]. The role of CMV’s immune-evading mechanisms in maintaining a proinflammatory milieu and its implications in diseases with chronic inflammation is an unfolding area of research interest [14]. Our group and others have studied the role CMV infection may play in the development of inflammatory conditions such as cardiovascular diseases [15], [16], [17], [18], [19]. These studies show, inter alia, that CMV induces mRNA expression of PAI-1 in endothelial cells, that CMV-induced effector T cells cause endothelial cell damage, that the virus induces thrombocyte activation and that CMV protein expression is highly prevalent in atherosclerotic plaques. The latter suggests that CMV is reactivated in the atherosclerotic plaques.

In this case-control study of a large cohort of patients with MI, we performed correlation analyses between CMV serology status and metabolic and inflammatory markers as well as apoB100 autoantibodies in blood samples collected three months after the initial MI.

## 2 Materials and methods

### 2.1 Study population

**Supplementary Fig. 1:**
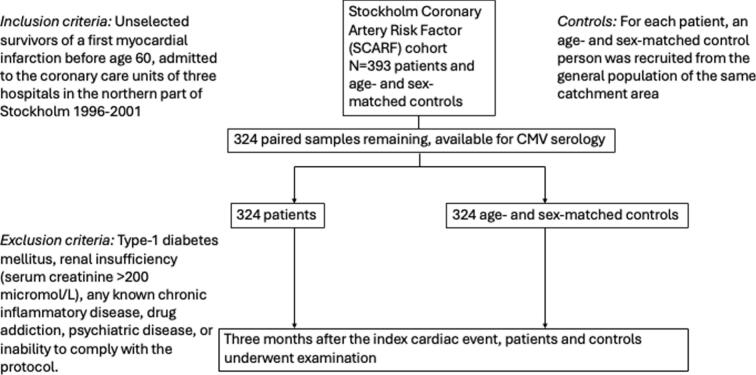


A total of 324 unselected survivors of a first-time MI below age 60 at three major hospitals in the region of Stockholm 1996–2001 were included, as described previously [20], [21], [22], [23], [24]. Age- and sex-matched controls were sampled from the general population. Written informed consent was obtained from each patient included in the study. The study protocol conforms to the ethical guidelines of the 1975 Declaration of Helsinki and has been priorly approved by the Institution's ethics committee on research on humans (Ethical permit Dnr 95–397, Dnr 2008/518–31). Three months after the initial MI, blood tests were taken at a follow-up visit and analysed for a vast range of parameters. Data from this cohort has been published previously [20], [21], [22], [23], [24]. Stored samples from 324 patients and corresponding controls were available for CMV serology analysis. A schematic of the study cohort is available as Supplementary Fig. 1. General characteristics for the included individuals are shown in Supplementary Table 1.

### 2.2 Biochemical analyses

A panel consisting of metabolic and inflammatory biomarkers were analysed at the Karolinska University Hospital laboratory and research laboratories at the Karolinska Institute, as described in earlier publications [20], [21], [22], [23], [24].

Serum samples, never used and stored at −80 °C without freeze thawing were analysed for CMV IgG with an in-house ELISA assay as previously described [25], [26], [27], [28], [29], manufactured by the Accredited Clinical Virology Laboratory, Karolinska University Hospital. We checked this ELISA assay in our laboratory by examining CMV-IgG negative (confirmed by Enzygnost Anti-CMV-IgG kit, Siemens Healthcare Diagnostics Products, Germany) and CMV-IgG positive (confirmed by CMV-IgG kit, Siemens) healthy blood donors. Results from the in-house ELISA confirmed the results from CMV-IgG kit (Enzygnost Anti-CMV-IgG kit, Siemens). In addition, a CMV-IgG quantification kit (CMV ELISA, IBL International, US) was used to evaluate positive controls used in our in-house ELISA at different dilutions according to the manufacturer’s instructions, to make a standard curve. In this ELISA assay, optical density values below 0.2 were considered negative, based on extensive analyses of sera from CMV-positive and CMV-negative blood donors, where mean optical density plus two standard deviations never exceeded 0.2 in seronegative reference sera. Paired samples from patients and matched controls were assessed on the same plate. For quality control, a standard curve was run on each plate.

### 2.3 Statistical analysis

Continuous variables are presented as median and inter quartile range (IQR). Categorical variables are presented as N (%). Levels of the analysed biomarkers were compared between cases and controls. Normality and lognormality tests were performed using Graphpad Prism 9 for each set of biomarkers **(**Supplementary Table 2**)**. Normally distributed sets of data were compared using unpaired *t*-test. Log transformations were performed for datasets with lognormal distributions. If data did not pass normality or lognormality tests, the Wilcoxon matched-pairs signed rank test was used to compare cases to controls.

Each set of biomarkers was analysed for association with CMV serostatus using two approaches. First, a binary variable for CMV serostatus was used to assess the effects of seropositivity, using the Mann-Whitney test. Second, non-parametric Spearman correlation analysis of levels of selected biomarkers and CMV IgG antibody levels in CMV positive individuals was performed, as CMV IgG antibody levels had a non-normal distribution.

## 3 Results

### 3.1 Basic characteristics

Differences in metabolic and inflammatory biomarkers between the cases and controls in the original study cohort of 387 case-control pairs have been previously described [20], [21], [22], [23], [24]. For the 324 pairs included in this study, general characteristics and cardiovascular risk factors are summarized in Supplementary Table 1. Statin use only occurred in the case cohort.

### 3.2 CMV serology

**Fig. 1:**
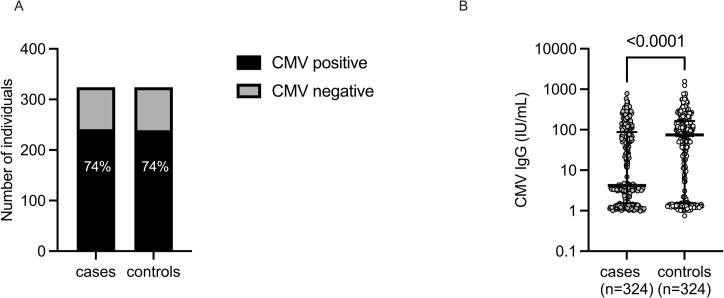
(A) CMV-IgG serology in 324 cases and 324 controls, respectively. (B) CMV-IgG levels in cases and controls, respectively. The error bars represent median with interquartile range (IQR).

The ELISA results showed that 241 (74 %) of 324 individuals in the case cohort and 239 (74 %) of 324 individuals in the control cohort, respectively, were positive for CMV IgG **(**Fig. 1**A)**. CMV IgG levels had a non-normal distribution. Median (interquartile range) CMV IgG level was 4.2 IU/ml (1.5–87.9) for cases and 74 IU/ml (2–163) for controls, respectively **(**Fig. 1**B)**, with cut-off level for positive CMV IgG at 1.65 IU/ml (OD > 0.2). Two outliers in CMV IgG levels were identified among the controls, measuring 1560 IU/ml and 1245 IU/ml, respectively. The difference in CMV IgG levels between cases and controls remained significant (p < 0.0001) upon removal of the outliers and their matching cases. The outliers with matching cases were kept throughout the study.

### 3.3 Impact of CMV on metabolic biomarkers

**Fig. 2:**
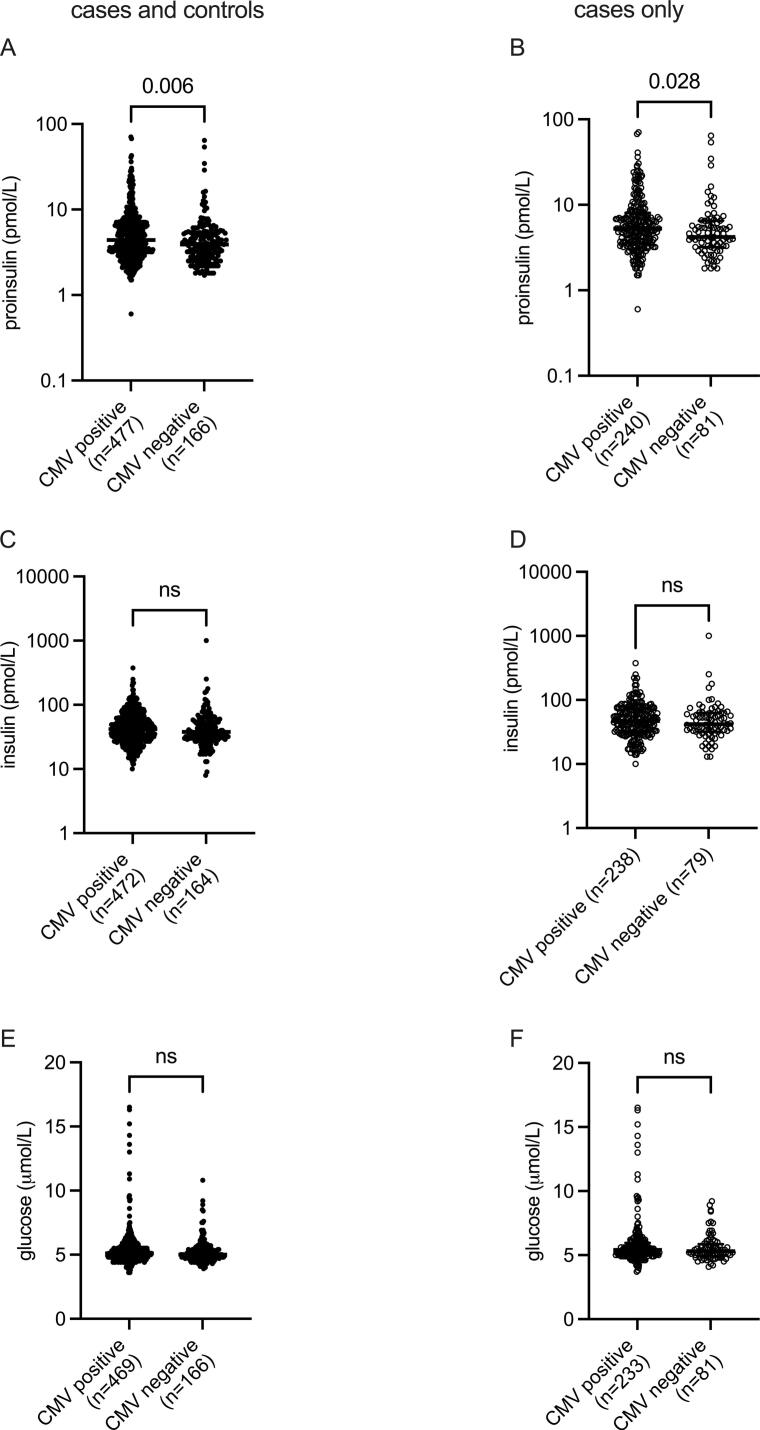
Levels of proinsulin (A,B), insulin (C,D) and glucose (E,F) in all CMV seropositive and –negative individuals in the study and the case cohort only, respectively. All data are represented as median and IQR.

**Supplementary Fig. 2:**
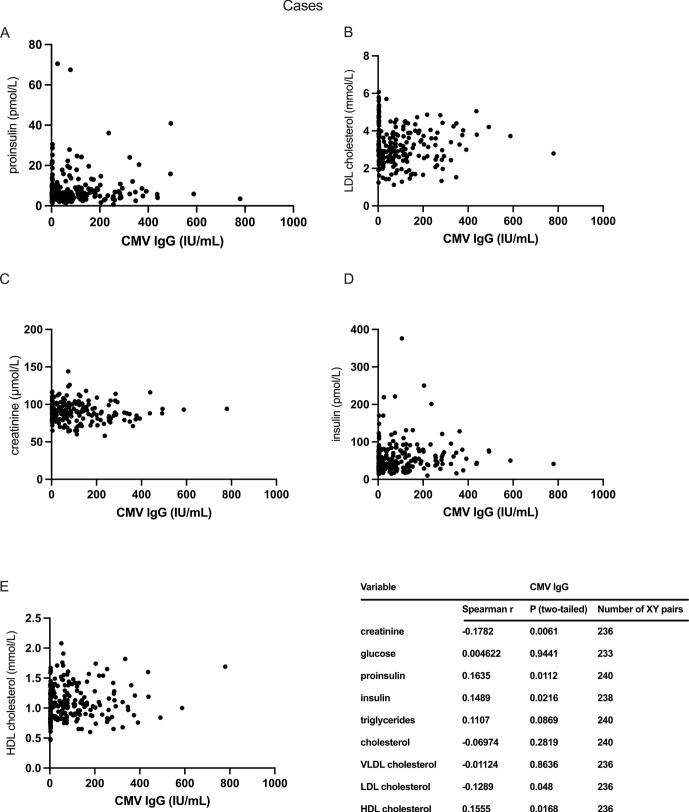


**Supplementary Fig. 3:**
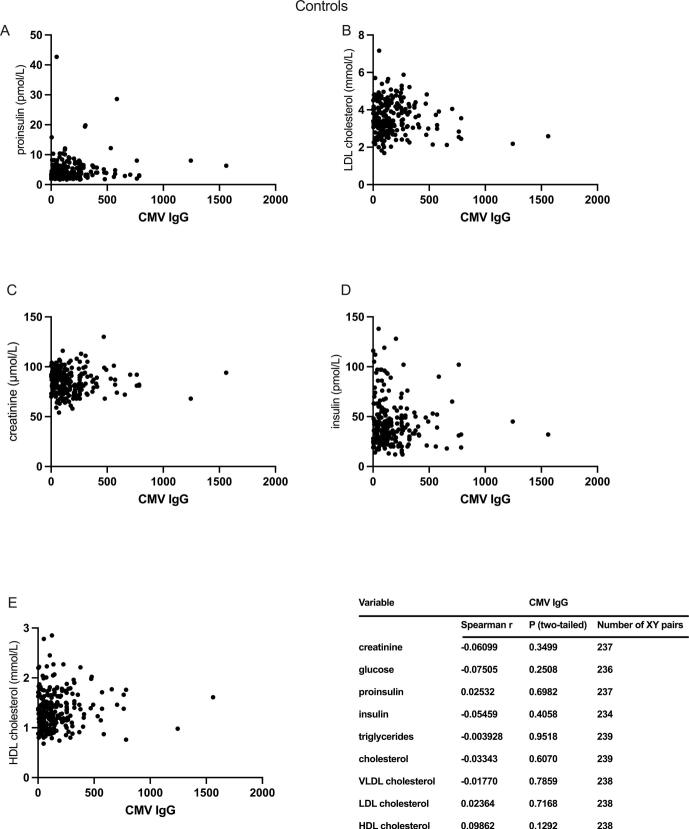


CMV seropositive individuals (both cases and controls) had higher proinsulin levels than the seronegative, a difference that remained significant in the case cohort **(**Fig. 2**A,B)**. A positive correlation was observed between CMV IgG and proinsulin levels in CMV seropositive MI patients; Spearman ρ = 0.1635, (*p* = 0.0112) **(**Supplementary Fig. 2A**)**, but not in CMV seropositive controls **(**Supplementary Fig. 3**A)**. Neither insulin levels **(**Fig. 2**C,D)** nor glucose levels **(**Fig. 2**E,F)** differed significantly between CMV seropositive and CMV seronegative individuals. Moreover, there was no significant correlation between CMV seropositivity and diabetes diagnosis (*p* = 0.1682, Fisher’s exact test). Of note, in the case cohort, 38 individuals had diagnosed diabetes mellitus. Only 10 individuals in the case cohort had elevated proinsulin levels (>28 pmol/L). Among these, seven had diagnosed diabetes, of which three were not treated with any glucose-lowering agents.

There were no significant differences in lipid levels between CMV seropositive and −negative individuals in either patient or control groups. There was a trend that CMV seropositive individuals (all individuals as well as the case cohort only) had lower HDL cholesterol than CMV seronegative individuals, however this was not statistically significant (data not shown). The difference in HDL cholesterol level between CMV seropositive and −negative individuals was completely abrogated in MI patients receiving statin treatment.

Against expectations, in CMV seropositive MI patients weak correlations were observed between CMV IgG and LDL cholesterol and between CMV IgG and HDL cholesterol **(**Supplementary Fig. 2B,**E)**. In all MI patients, there was also a weak negative correlation between CMV IgG levels and creatinine **(**Supplementary Fig. 2C**)**. No such correlations were found in CMV seropositive controls **(**Supplementary Fig. 3), suggesting that any potential link between CMV IgG levels and cholesterol levels are dependent on other processes in MI patients.

### 3.4 Impact of CMV on inflammatory biomarkers

**Fig. 3:**
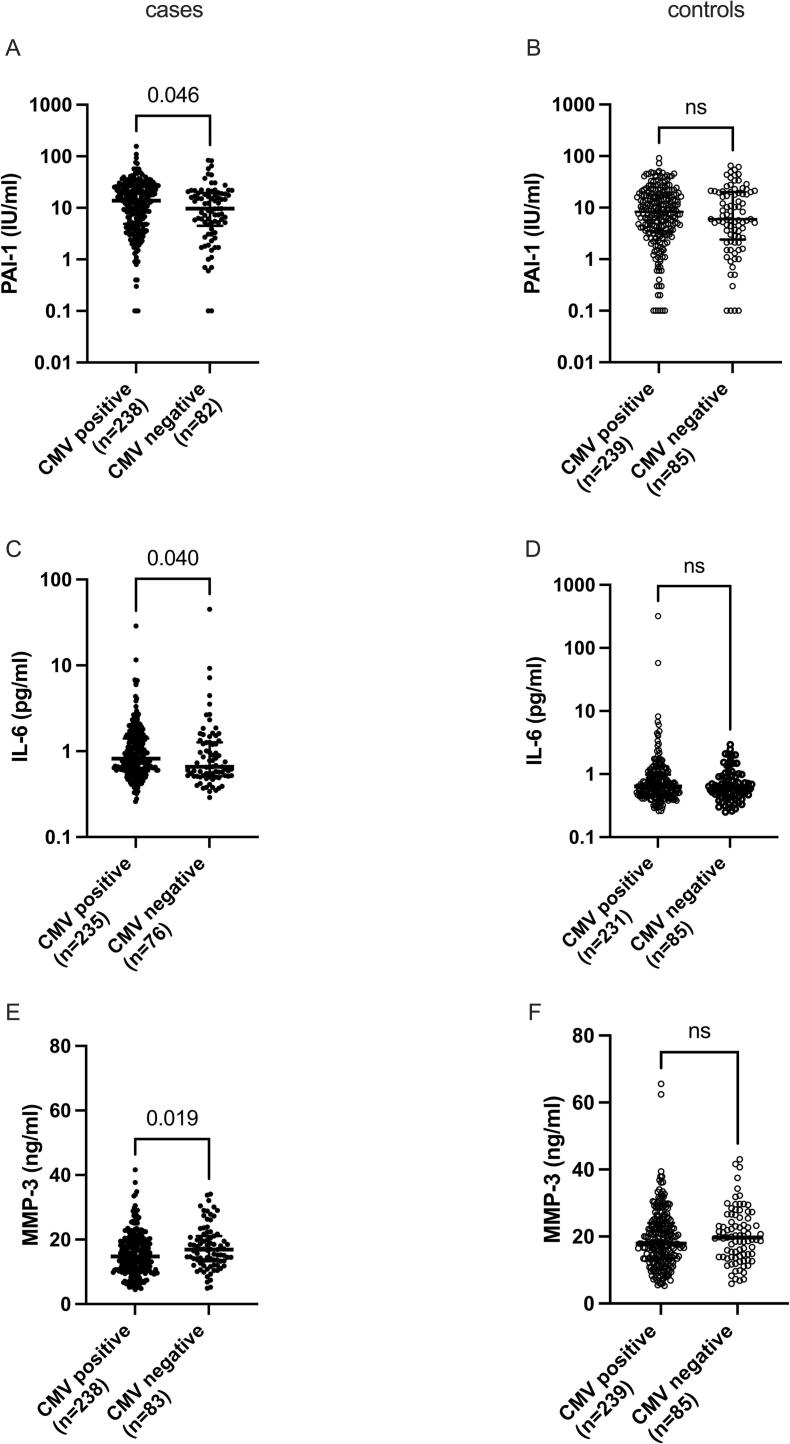
Inflammatory markers that differed significantly, depending on CMV serostatus, in cases and controls, respectively. Bars represent median and IQR.

CMV seropositive cases showed significantly increased levels of PAI-1 (p = 0.046) and IL-6 (p = 0.040), and decreased levels of MMP-3 (p = 0.019) **(**Fig. 3**A, C, E)**, compared to CMV seronegative cases. Such differences were not significant for controls (Fig. 3**B, D, F)**. Notably, there was a vast range of IL-6 levels in the control cohort **(**Fig. 3**D)**, indicating unaccounted factors in the control group. Multivariate analysis of CMV IgG levels and PAI-1, IL-6, and MMP-3 in CMV positive individuals of the case cohort showed a weak correlation between CMV IgG levels and PAI-1 (*p* = 0.0059, Spearman’s ρ 0.1778), but no correlation between CMV IgG levels and IL-6, and MMP-3, respectively.

### 3.5 Impact of CMV on apoB100 autoantibodies

**Fig. 4:**
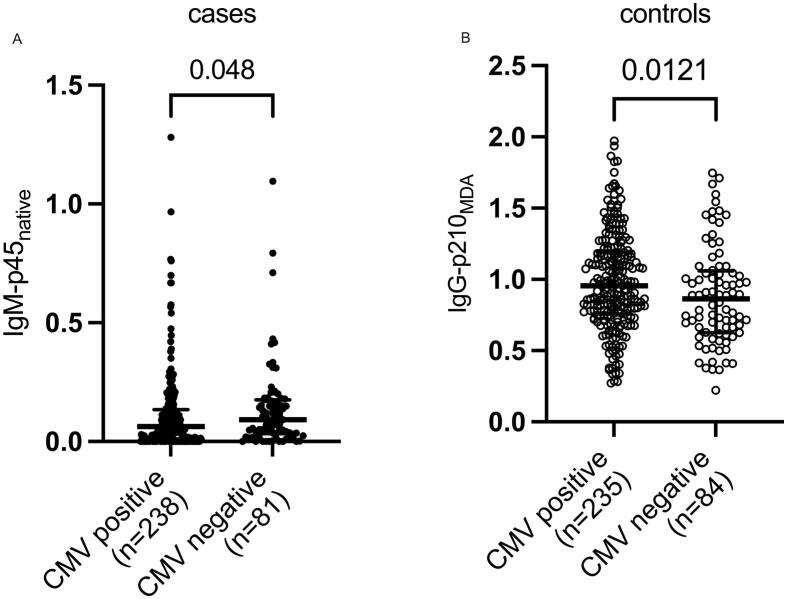
ApoB100 autoantibodies that differed significantly, depending on CMV serostatus, represented as median and IQR.

The differences between cases and controls regarding apoB100 autoantibodies have been described previously [30]. A comparison between apoB100 autoantibody levels in CMV seropositive and −negative individuals in the case and the control cohorts, respectively, revealed significantly lower IgM-p45_native_ levels in CMV positive MI patients **(**p = 0.048**,** Fig. 4**A)**. In the control cohort, the only significant difference in autoantibody levels between CMV seropositive and −negative individuals was noted for IgG-p210_MDA_ levels (p = 0.012), which were higher in CMV seropositive individuals **(**Fig. 4**B)**.

## 4 Discussion

In the present study, we have investigated the potential impact of being a carrier of CMV on metabolic and inflammatory markers that set cases apart from controls in a study of post-MI before the age of 60. A total of 324 pairs of MI patients and age- and sex-matched controls were included and sampled 3 months after the event. The methods used to generate the datasets concerning metabolic and inflammatory biomarkers have today, in some cases, been replaced by newer methods. ELISA was used to measure several biomarkers and cytokines, such as insulin, proinsulin and IL-6, using commercially available kits. Although these kits are still in production, electrochemiluminescense immunoassay (ECLIA) has advantages over ELISA in terms of sensitivity and speed due to the possibility of running multiplex assays [31], [32]. However, this does not affect the groupwise comparisons within our study, as all analyses were performed with the same method. A couple of important aspects should be noted in the interpretation of the results and are discussed below: the timing of the sampling, the measuring of IgG levels alone, and the interplay between CMV infection and inflammatory markers in this cohort of MI patients below the age of 60.

Although CMV prevalence was equal in cases and controls, CMV IgG levels were significantly lower in cases compared to controls. Our results support those of Adler et al. [33] and Hamilton et al. [34], who find no evidence of CMV seropositivity as a major risk factor for atherosclerosis or cardiovascular disease (CVD), respectively. Contrary to these findings, higher CMV IgG levels were shown to be associated with incident ischemic heart disease in a population-based study by Gkrania-Klotsas et al [35]. Blum et al. [36] also found higher CMV IgG antibody titers in patients with coronary artery disease, in a study including 65 patients (76 % men) aged 40 to 75 years. Simanek et al. [37] found that CMV seropositivity was predictive for both cardiovascular mortality as well as all-cause mortality. The contribution of CMV infection to CVD has also been shown in several other studies, as discussed in several reviews [38], [39]. The discrepancy in our results with earlier published results may be explained by the difference in the sampling timepoint –three months after MI. We studied a younger patient cohort that may depict different pathophysiological mechanisms of atherosclerosis and MI that may be affected by genetic risk factors more prominent in the younger patients (maximum 60 years, mean age 53 years), who were mainly men (85 %). At the time of sampling, 92 % of our MI cases were taking acetylsalicylic acid (ASA), which may affect B cell function and antibody responses [40], [41]. Still, many inflammatory biomarkers were higher in the cases compared to the controls. Of these, both TNF-⍺ and IL-6 have been shown to drive reactivation of CMV [42], [43], [44], but ASA has also been shown to inhibit CMV replication [45]. As CMV is reactivated by inflammation and dependent on COX-2 expression, suppressing inflammation with ASA may lead to a lower rate of reactivation. Here, we however found no significant difference in CMV IgG levels in patients with or without ASA treatment. It is not known if CMV seroprevalence in Sweden have changed during the past two–three decades, but a recent longitudinal analysis of the overall CMV seroprevalence in Germany for the past three decades indicated that a plateau had been reached [46]. Seroprevalence tends to decrease with higher economic standards and increase with age [47]**.** Since Germany is comparable to Sweden in terms of life expectancy and GDP per capita [48], our data may be applicable to today’s immune status. This assumption is further supported by our own experience of analysing serum samples for HCMV seroprevalence over three decades in Swedish adults, observing remarkably constant seroprevalence levels as observed in this patients cohort and controls.

CMV reactivation is likely a dynamic event that can occur at different intensities, as hypothesized by Collins-McMillen and Goodrum [49]. Reactivation may not always lead to viremia, which was used as a measure of virus reactivation in a study of kidney transplant patients, who had CMV antibody titers that were modulated by the frequency and intensity of virus reactivations [50]. This may explain why the negative findings of the present study do not contradict molecular biological evidence of CMV infection found in atherosclerotic lesions in numerous other studies, as discussed by Du et al. [39].

As noted before [38], epidemiologic studies have generally used the detection of CMV IgG antibody to determine the presence of CMV infection. However, there are many unknown aspects regarding individual immune responses to CMV other than IgG levels, such as individual T- and B-cell function, as well as antibody avidity and neutralizing effect. Higher levels of CMV IgG may in fact reflect more robust immune functions and higher ability to control CMV reactivation and replication. CMV IgG kinetics have been studied in the setting of CMV infection in transplant patients [51], [52] and in pregnant women and their children, but much remains unknown about CMV IgG kinetics in immunocompetent individuals. Importantly, it is not known whether CMV IgG levels correlate to grade of CMV infection, present locally in the inflamed tissue or systemically, or whether higher antibody titres reflect a better immune response and control of a lingering infection.

The significant differences in metabolic markers shown between cases and controls reflected known risk factors for cardiovascular disease. CMV seropositive individuals had higher proinsulin levels than the CMV seronegative in both cases and controls, and a weak correlation between CMV IgG level and proinsulin was found. Proinsulin levels are increased by IL-6 [53], and IL-6 levels were higher in CMV positive patients. While it is known that CMV infection disrupts the normal intermediary metabolism on cellular level by modifying glucose and glutamine metabolism in a manner reminiscent of tumor cells [54], there is no established association between active CMV infection and insulin or glucose levels *in vivo*. Our present study also failed to show a correlation between CMV serostatus and insulin or glucose levels. Proinsulin could not be used as a pseudomarker for diabetes in this study, as most individuals with diabetes did not have elevated proinsulin levels. Previous studies have shown that CMV induces the production of IL-6 [55], [56], [57]. Blankenberg et al [58] showed that in patients with coronary artery disease, CMV-seropositivity independently correlates with elevated IL-6 levels and that CMV infection predicts cardiac mortality in patients with elevated IL-6 levels. Of note, the association with CMV seropositivity with future cardiac mortality was lost in patients without an inflammatory response and, in conclusion, these data support the hypothesis that the effects on atherosclerosis and myocardial infarction related to CMV infection are likely mediated via an underlying inflammatory response. While CMV can drive inflammatory processes, the virus is also reactivated by inflammation, demonstrating a close relationship between inflammation and CMV activity.

Earlier clinical studies suggest that high levels of autoantibodies targeting the p45 and p210 amino acid sequences on apoB100 peptides in oxidized LDL have a protective role in the development of cardiovascular disease [7]. Experimental studies have demonstrated that presence of these autoantibodies indicate a cell-mediated immune response against oxidized LDL in a way that is atheroprotective [59], [60]. As CMV is known to interfere with the cellular immune response in numerous ways, CMV infection may affect the individual ability to form apoB100 autoantibodies. Here, no direct correlation was determined between CMV seropositivity and presence of apoB100 autoantibodies. IgM-p45_native_ was significantly lower in the case cohort compared to controls, and in the case cohort, CMV seropositive individuals had lower IgM-p45_native_ levels than the CMV seronegative. However, the difference in IgM-p45_native_ levels between CMV seropositive and seronegative controls was not significant (data not shown).

As atherosclerosis is an inflammatory process, proinflammatory markers are expected to be elevated in patients with MI. It has been shown that CRP correlates with the severity of coronary heart disease [61]. In the same study, IL-1β levels were increased in patients at one and six months after MI, compared to baseline. Simanek et al. found that CMV seropositive individuals with CRP levels ≥ 0.3 mg/dL had a 29.5 % higher risk for CVD-related mortality compared to CMV seropositive individuals with lower CRP levels [37]. Another study of a general adult population showed significant positive association between intensity of anti-CMV IgG responses and vascular injury biomarkers soluble intercellular adhesion molecule 1 (sICAM-1) and soluble vascular cell adhesion molecule 1 (sVCAM-1), but not CRP and serum amyloid A (SAA) [37]. In agreement with this, we did not find any correlation between CMV seropositivity and CRP or SAA in the patient cohort analysed.

In a mouse model, cardiac PAI-1 as well as PAI-1 antigen in plasma increased post MI, and expression of TNF-⍺ and TGF-β increased in cardiac tissue post MI [62]. These results support our findings of increased proinflammatory markers three months after MI, compared to the control cohort. In the case cohort, CMV positive individuals had higher levels of PAI-1 and IL-6 than the CMV negative individuals. CMV induces PAI-1 expression in endothelial cells [63] and adipocytes [64]. In CMV positive individuals, PAI-1 levels correlated with CMV IgG levels in the case cohort, but not in the controls. We observed no correlation between CMV IgG levels and IL-6 in either cohort, nor overall in all study subjects, such as previously reported by others [65], [66], even though the CMV seropositive individuals tended to have higher IL-6 levels than the CMV seronegative in the case cohort as well as the control cohort. Importantly, a wider range of IL-6 levels was measured in the CMV seropositive compared to CMV seronegative controls, indicating inflammatory activity of unknown reasons among seropositive controls. Indeed, the control subject with the highest IL-6 level was a CMV seropositive male with several risk factors for CVD; smoking, a sedentary occupation and high BMI. The interpretation of the possible role of CMV infection in inflammation is further limited by the presence of co-morbidities associated with inflammation [67], [68]. In the case cohort, co-morbidities such as diabetes mellitus type 2 and hyperlipidemia were much more common compared to the control cohort. A history of autoimmune disease was not documented in the cohorts. During the COVID-19 pandemic, proinflammatory cytokines, in particular IL-6, were shown to be elevated in cases of severe disease. Although increased levels of IL-6 have been reported in patients with Long COVID [69], there is to our knowledge no evidence of elevated levels of baseline IL-6 in the general population following COVID. Therefore, we think that COVID would not modify our findings if repeated in samples collected at the present time.

MMP-3 levels were lower in cases compared to controls, as well as lower in CMV seropositive compared to seronegative individuals in the case cohort. MMP-3 is present in atherosclerotic plaques and increased MMP-3 levels have been reported as an individual cardiovascular risk factor [70]. MMP-3 is mainly expressed by macrophage-derived foam cells in unstable plaques [71] and aortic smooth muscle cells (AoSMC). PAI-1 can indirectly affect MMP expression [72]. On the other hand, CMV infection induced MMP-3 in AoSMC *in vitro* [73], in contrast to our findings.

In addition to ASA, lipid lowering and oral anti-diabetic drugs were prescribed to 32 % and 7 % of the patients, respectively. This study was conducted on samples collected 1996–2001. Since then, secondary prevention of ischemic heart disease has changed to include dual anti-platelet therapy (DAPT), which may have further anti-inflammatory properties [74], [75], and statins for all patients [74], Immunomodulatory properties of statins [5] as well as metformin [76], [77] are subject to mounting interest. While MMP-3 and MMP-9 levels do not appear to be affected by statin therapy [78], other inflammatory markers measured in this study may have been affected. Further, the study is biased towards patients that have survived MI. The biomarkers included in this study were not measured for all the patients, leading to missing values that may have affected the sample size.

In conclusion, we provide evidence that CMV positive post-MI patients had higher IL-6 and PAI-1 levels, and lower MMP-3 levels, a pattern that may affect their thromboembolic state, schematically summarized in Supplementary Fig. 4. Patients had lower levels of CMV IgG than controls, which may indicate a lower immune response to CMV and hence higher CMV activity in patients, or a possible effect of the post-MI treatment including ASA and statins lowering CMV activity. We suggest that further studies aiming to investigate the role of CMV infection in chronic inflammation should employ a more granular view of the immune response to CMV by also including measurements of B and T lymphocyte function. In lack of studies on how CMV IgG levels fluctuate in health and disease and what impact this has on immune control of CMV, further investigations of correlations between CMV serology and different biochemical markers are unlikely to enhance our understanding of a potential role of CMV infection in atherosclerosis and MI.

## 5 Author contributions.

The study design was conceived by C S-N and AR, who also contributed to preparation of the manuscript. PL recruited study subjects and participated in the conceptualization of the SCARF study cohort. Sample collection was coordinated by AS, who also contributed to laboratory analyses. Data analysis, preparation of tables and figures and drafting of the manuscript was performed by XX. All authors read and approved of the final manuscript.

## 6 Financial support

This study was supported by The Swedish Medical Research Council Grant # 2022-02724.

## CRediT authorship contribution statement

**Xinling Xu:** Writing – review & editing, Writing – original draft, Visualization, Formal analysis, Data curation. **Angela Silveira:** Writing – review & editing, Data curation. **Pia Lundman:** Writing – review & editing, Data curation. **Afsar Rahbar:** Writing – review & editing, Supervision, Data curation, Conceptualization. **Cecilia Söderberg-Nauclér:** Writing – review & editing, Supervision, Methodology, Funding acquisition, Conceptualization.

## Declaration of competing interest

The authors declare that they have no known competing financial interests or personal relationships that could have appeared to influence the work reported in this paper.

## Figures and Tables

**Supplementary Fig. 4:**