# Supplementary material for: Enhanced levels of IL-6 and PAI-1 and decreased levels of MMP-3 in cytomegalovirus seropositive patients with prior myocardial infarction
Source: Int J Cardiol Heart Vasc. 2024 Dec 2;56:101570. doi: 10.1016/j.ijcha.2024.101570 (PMC11650320; doi:10.1016/j.ijcha.2024.101570)
Supplement: Supplementary Data 1 [file mmc1.docx]

**Supplementary Table 1.** General characteristics and cardiovascular risk factors from the participants of the study, from whom we had samples to analyse CMV serology (n=324 postinfarction patients and 322_a_ matched controls).

| **Risk factor** | **Cases (N=324)** | **Controls_a_ (N=322)** | **P-value** |
| --- | --- | --- | --- |
| Age, years; median (IQR) | 54.04 (7.43) | 54.07 (7.54) |  |
| Sex; % male:female | 85:15 | 85:15 |  |
| Smoking; N (%) |  |  |  |
| Never | 84 (26) | 129 (40) | 0.0002_c_ |
| Previous smoker | 182 (56) | 121 (38) | <0.0001_c_ |
| Current smoker | 58 (18) | 72 (23) | 0.1701_c_ |
| Alcohol, g/week;  median (IQR) | 60 (112) | 99 (128) | <0.0001_d_ |
| BMI, kg/m^2^; median (IQR) | 26.8 (5) | 25.6 (4) | <0.0001_d_ |
| Diabetes mellitus type II;  N (%) | 38 (12) | 0 | <0.0001_c_ |
| Hypertension_b_; N (%) | 112 (5) | 20 (6) | <0.0001_c_ |
| Family history of ischemic heart disease; ; N (%) | 136 (42) | 64 (20) | <0.0001_c_ |
| Family history of hypertension; N (%) | 116 (36) | 104 (32) | 0.3611_c_ |
| Family history of diabetes mellitus; N (%) | 38 (12) | 0 | <0.0001_c_ |
| Family history of hyperlipidemia; N (%) | 66 (20) | 25 (8) | <0.0001_c_ |
| **CMV serostatus** | **Cases** | **Controls** |  |
| Positive; N (%) | 241 (74) | 239 (74) |  |
| **Drug class**; N (%) | **Cases** | **Controls** |  |
| Acetylsalicylic acid | 299 (92) | 3 (1) |  |
| Beta blocker | 306 (95) | 6 (2) |  |
| Statin | 104 (32) | 0 |  |
| ACE inhibitor | 108 (34) | 1 (0.3) |  |
| Anti-diabetic (oral) | 24 (7) | 0 |  |
| Nitrate | 270 (83) | 0 |  |
| Diuretic | 56 (17) | 0 |  |
| Hormonal treatment | 14 (4) | 18 (6) |  |

BMI, body mass index. Values are presented as median (interquartile range, IQR), or N (%) subjects in each group. _a_Data on lifestyle factors and health history missing in two controls. _b_Including individuals using antihypertensive therapy. _c_Fisher’s exact test. _d_Wilcoxon test
