# Supplementary material for: Enhanced levels of IL-6 and PAI-1 and decreased levels of MMP-3 in cytomegalovirus seropositive patients with prior myocardial infarction
Source: Int J Cardiol Heart Vasc. 2024 Dec 2;56:101570. doi: 10.1016/j.ijcha.2024.101570 (PMC11650320; doi:10.1016/j.ijcha.2024.101570)
Supplement: Supplementary Data 2 [file mmc2.docx]

**Supplementary Table 2.** Summary of results from normality and lognormality tests.

|  | Passed normality test?_a_ | |
| --- | --- | --- |
| Biomarker | **Cases** | **Controls** |
| Creatinine | No | Normal |
| Glucose | No | No |
| Proinsulin | No | No |
| Insulin a | No | No |
| Triglycerides | No | Lognormal |
| Cholesterol | Lognormal | Lognormal |
| VLDL cholesterol | Lognormal | No |
| LDL cholesterol | Normal | Normal |
| High density lipoprotein (HDL) cholesterol | No | Lognormal |
| Cystatin C | Lognormal | Lognormal |
| IgM-p45_native_ | No | No |
| IgG-p45_native_ | No | No |
| IgM-p45_MDA_ | No | No |
| IgG-p45_MDA_ | No | No |
| IgM-p210_native_ | Normal | No |
| IgG-p210_native_ | Normal | Lognormal |
| IgM-p210_MDA_ | No | Normal |
| IgG-p210_MDA_ | Normal | No |
| CRP | Lognormal | Lognormal |
| Plasminogen activator inhibitor (PAI)-1 | No | No |
| Fibrinogen | No | No |
| Serum amyloid A (SAA) | No | No |
| Tumor necrosis factor (TNF)-α | No | No |
| Matrix metalloproteinase (MMP)-3 | No | No |
| MMP-9 | No | Lognormal |
| Interleukin (IL) -1a | No | No |
| IL-1b | No | No |
| IL-2 | No | No |
| IL-4 | No | No |
| IL-6 | No | No |
| IL-10 | No | No |
| IL-18 | No | Lognormal |
| Epidermal growth factor (EGF) | Lognormal | Lognormal |
| Vascular endothelial growth factor (VEGF) | No | Lognormal |
| Monocyte chemoattractant factor (MCP)-1 | No | No |
| Factor VIIa (FVIIa) | Lognormal | Lognormal |

_a_If yes, data is distributed normal or lognormal
